# Supplementary figures and images for: G4SNVHunter: An R/Bioconductor Package for Evaluating SNV-Induced Disruption of G-Quadruplex Structures Leveraging the G4Hunter Algorithm
Source: PLoS Comput Biol. 2025 Aug 18;21(8):e1013368. doi: 10.1371/journal.pcbi.1013368 (PMC12373273; doi:10.1371/journal.pcbi.1013368)

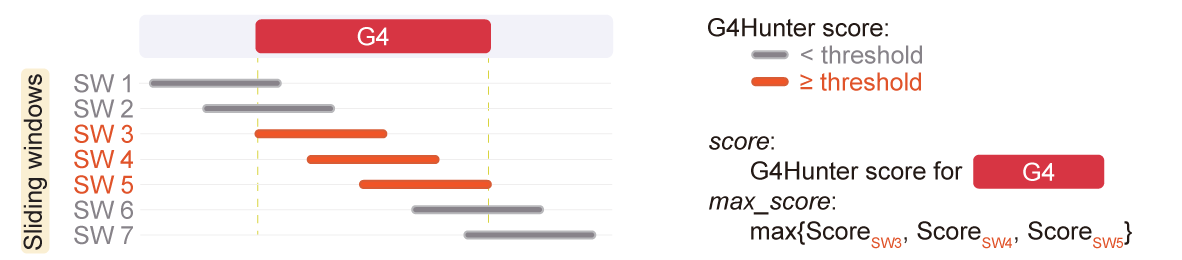

Supplement: S1 Fig — In brief, the program calculates the G4Hunter score within a fixed-length sliding window (e.g., 25 bp). Windows with scores above a threshold (e.g., 1.5) are marked as positive windows. Overlapping positive windows are merged into larger G4 regions, and the maximum score within each region is taken as the maximum window score, reflecting the highest structural formation propensity within the G4 region. (S1_Fig.TIF) [file pcbi.1013368.s001.tif]

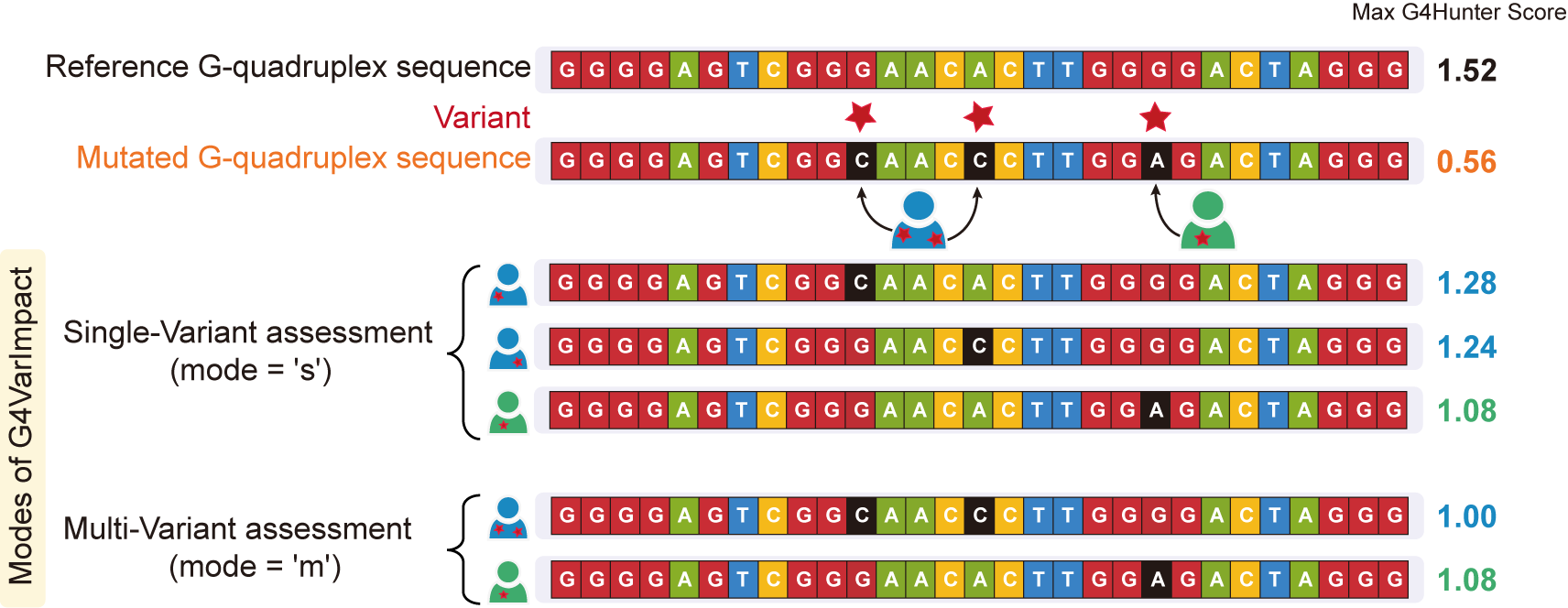

Supplement: S2 Fig — The top is an example G4 sequence with a maximum G4Hunter score of 1.52. Suppose there are three possible variants located in the G4 region, originating from sample A (blue) and sample B (green). Variant positions are indicated by stars; mutant nucleotides are shown in black. In the single-variant mode, sample information is ignored; therefore, G4SNVHunter will assess the impact of these three variants on that G4 separately, resulting in three evaluation records, each having the maximum G4Hunter score after the introduce of a single variant into the G4 sequence (G > C: 1.28; A > C: 1.24; G > A: 1.08). In contrast, in the multi-variant mode, G4SNVHunter will make calculations on a sample-by-sample basis. Since both G > C and A > C variants originate from sample A, the program will calculate the combined effect of their impact on the formation of that G4 (maximum G4Hunter score drops to 1.00). (S2_Fig.TIF) [file pcbi.1013368.s002.tif]

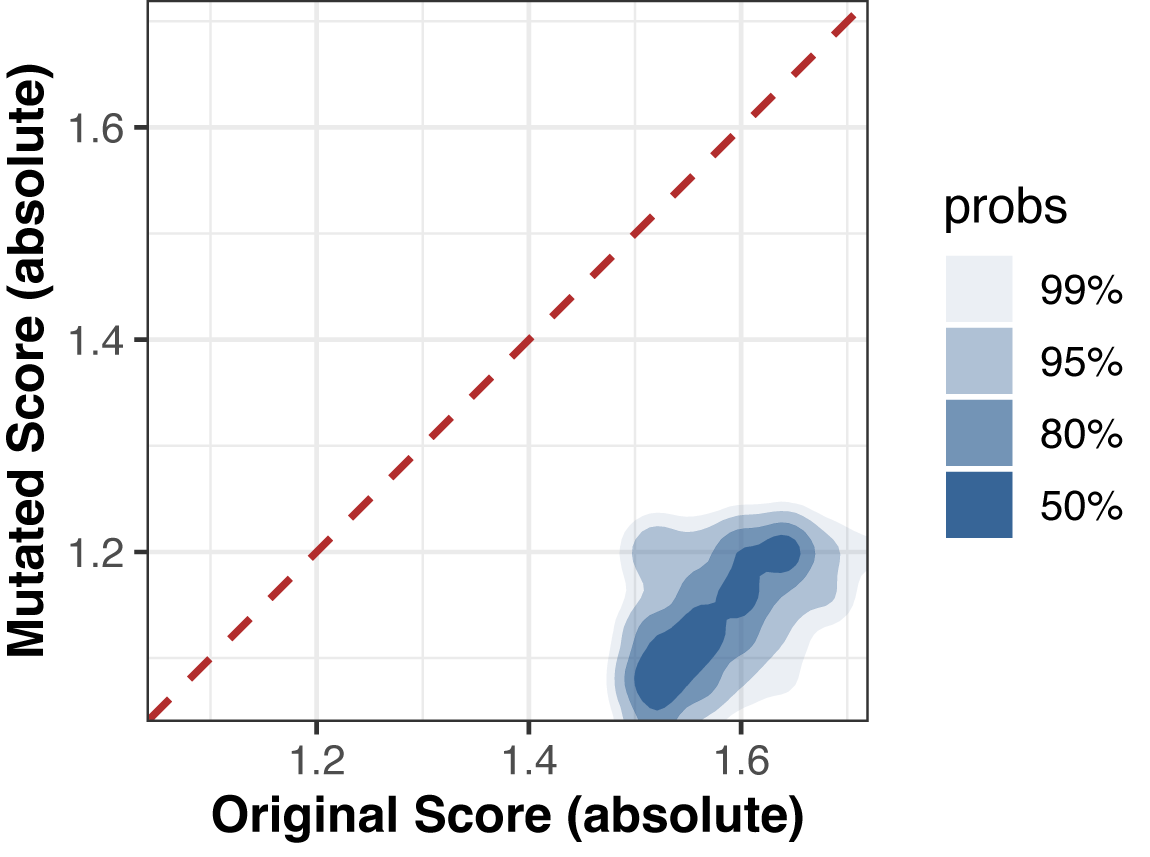

Supplement: S3 Fig — These G4s have formation propensity scores > 1.5 before variation and < 1.2 after; G4s with increased or mildly reduced propensity were excluded. (S3_Fig.TIF) [file pcbi.1013368.s003.tif]

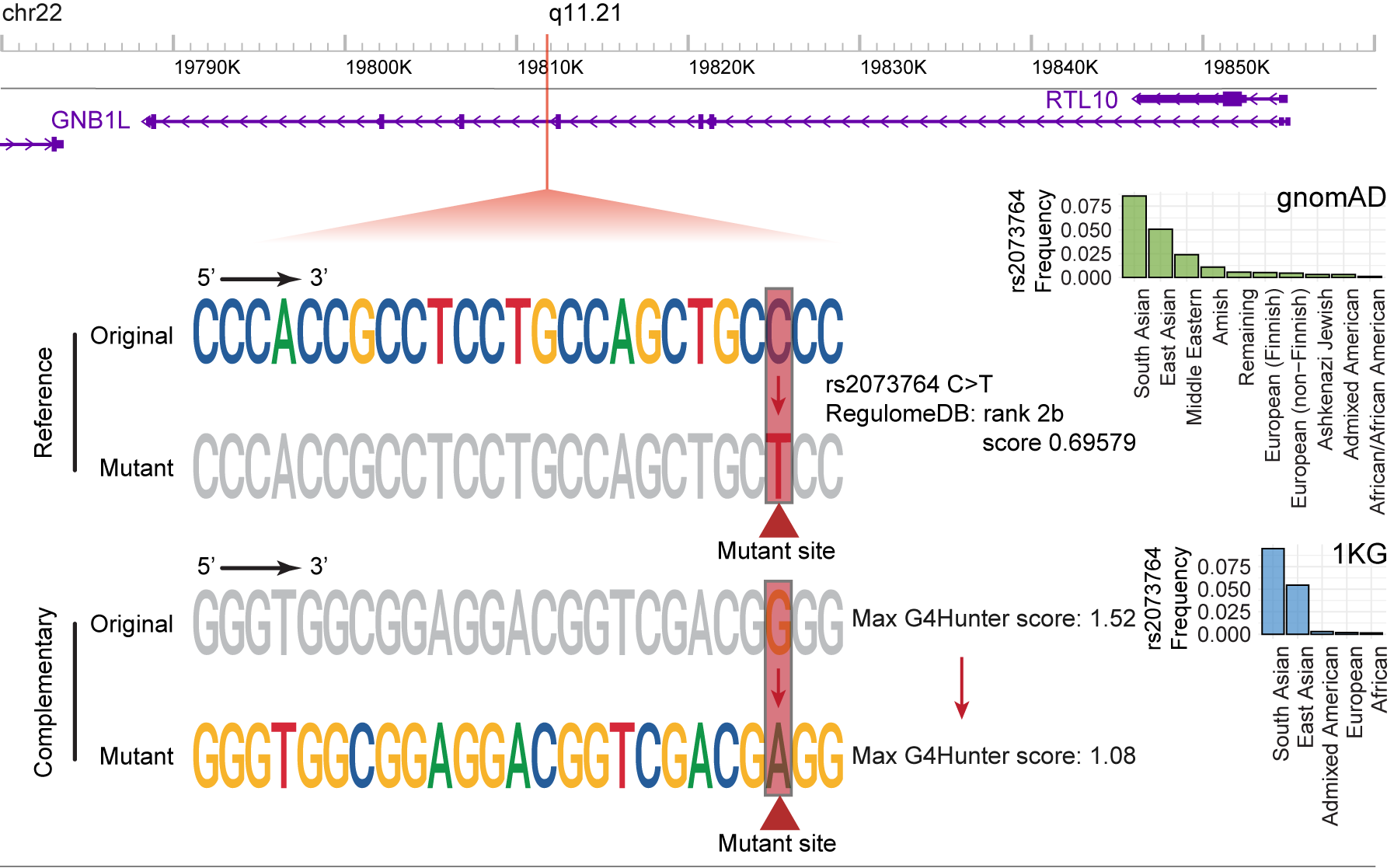

Supplement: S4 Fig — The variant (rs2073764) is located in the intronic region of the GNB1L gene and can reduce the formation score of the G4 on the complementary strand from 1.52 (stable state) to 1.08 (unstable state). This variant was found to be associated with non-syndromic cleft lip with cleft palate in a study based on the Chinese Han population. The bar chart on the right shows the frequency of this variant in Asian, European, African, and American populations in the gnomAD and 1000 Genomes Project databases (data source: https://gnomad.broadinstitute.org/variant/22-19811887-C-T?dataset=gnomad_r4, accessed October 2024). (S4_Fig.TIF) [file pcbi.1013368.s004.tif]
