# Supplementary material for: G4SNVHunter: An R/Bioconductor Package for Evaluating SNV-Induced Disruption of G-Quadruplex Structures Leveraging the G4Hunter Algorithm
Source: PLoS Comput Biol. 2025 Aug 18;21(8):e1013368. doi: 10.1371/journal.pcbi.1013368 (PMC12373273; doi:10.1371/journal.pcbi.1013368)
Supplement: Attachment — Submitted filename: Response_to_Reviewer_Comments.pdf [file pcbi.1013368.s009.pdf]

Reviewer #1:

General comments: In this manuscript, the authors present an R/Bioconductor package for evaluating the impact of single nucleotide substitutions on G-quadruplex formation.

The package functions as a wrapper for the G4Hunter algorithm and includes a convenient feature for assessing genome-wide variations. However, I have concerns about its overall usefulness to the community due to its limited functionality.

Response: Thank you very much for reviewing our manuscript and for your valuable comments. In response to your concerns about functionality, we modified the package accordingly and added several features to further enhance its usefulness and applicability to the community. Please see our detailed point-by-point responses below.

Comment: Lack of VCF file support: It does not appear that the package accepts variants in VCF format, which is the standard format for variant representation. The inability to parse VCF files is a significant drawback.

Response: Thank you for pointing this out. In the latest version of our package, we added support for VCF files. In addition, considering that MAF (Mutation Annotation Format) is also a commonly used mutation data format, we extended compatibility to include MAF files as well. Users can easily load variant files and perform subsequent analysis through our dedicated functions. We added a description of this function in the manuscript.

"*G4SNVHunter requires users to provide a collection of variants of interest and load them into the environment in the form of a GRanges object using the loadVariant function. Both Variant Call Format (VCF) and Mutation Annotation Format (MAF) are supported.*" (line 111 to 113)

Comment: Handling of small indels (1–4 bp): While indels are less common than point mutations, they can have a stronger impact on G-quadruplex formation. Given that handling small indels is not fundamentally different from handling point mutations, I would expect the package to support them.

Response: We fully agree with the suggestion. In response, we refactored the underlying code of the relevant functions. In the latest version, the software now supports multiple types of small-scale variants, including single base variants (SNVs), small

insertions/deletions (indels), and multinucleotide variants (MNVs), thus significantly improving the completeness and usefulness of the tool.

We referenced this revision in the manuscript. For example,

"*Here, we introduce an R/Bioconductor package called G4SNVHunter, which is based on the G4Hunter algorithm [4] and specifically designed to identify SNVs, also applicable to other small-scale variants like indels (insertions and deletions), that may impair the formation of G4 structures.*" (line 66 to 68)

Comment: Speculative functional inference of SNPs: The manuscript suggests that two variants (in ATP1B3 and GNB1L) may be functionally relevant, but this claim appears purely speculative. The null hypothesis is that these variants are simply random genomic mutations. While it is reasonable to use these examples to demonstrate the package's application, any claims about functional significance should be supported by existing functional genomic data. For instance, mapped promoter regions (e.g., from ChIP-seq) in relevant cell types should be referenced for ATP1B3. Additionally, the GNB1L variant is located in an intron, and the rationale for its functional relevance is unclear. Including this variant in the abstract overstates its significance, as the authors provide no evidence of its effect on the gene.

Response: Accordingly, we made substantive revisions to the relevant content to address and answer your concern:

(1) **Updated Dataset and Regulatory Potential Assessment.** We replaced the originally used dataset with a more comprehensive archaic introgressed variant dataset from Neanderthals and Denisovans released by Dr. Akey's group (Browning et al., 2018, Cell) to improve both the representativeness and comprehensiveness of our analysis. Based on this, we used the RegulomeDB scores to systematically assess the regulatory potential of variants that can impact the formation propensity of G4s. This scoring system annotates and prioritizes variants based on epigenomic features such as chromatin accessibility and transcription factor binding. Our results show that compared to matched randomly selected archaic variants with similar base composition and number, these G4-impacting archaic variants have higher RegulomeDB scores. Please see Figure 2C for refer.

(2) **Prior Work Supporting Functional Consequences of G4 Variants**. In fact, we and other researchers have previously reported that some G4-associated variants may possess regulatory potential (e.g., PMID: 39183303; PMID: 34001600). In another study from our group (PMID: 37660977), we experimentally validated the impact of variants on

G4 structure formation in genes encoding cytochrome P450 enzymes. We later realized that some of these variants are of archaic human origin and are known to have functional consequences.

(3) **Revision to Avoid Overinterpretation.** Incorporating your suggestions, we comprehensively revised the relevant manuscript sections, including a rewrite of the abstract section. We retained the objective description of the analytical results, but removed speculative statements of potential functionality to ensure that the manuscript focuses on the method demonstration itself and avoids over-interpretation. For example, in the abstract, we changed the wording to (we provide objective descriptions without over-interpretation):

"To showcase the functionality of the G4SNVHunter package, we analyzed the Neandertal and Denisovan archaic introgressed variants detected by the Sprime software, and identified approximately 5,800 variants located within G4 regions, among which around 230 may impair G4 structure formation propensity." (line 26 to 29)

Another two examples are (we do not make regulatory inferences here, but rather state a fact or outline what can be tested in future research):

"*By querying RegulomeDB, this variant was found to have high regulatory potential (RegulomeDBV2.2 score: 0.98; range: 0-1), suggesting functional relevance. In fact, the impact of Neandertals on the modern human immune system has long been a topic of considerable research interest [17, 18]. We here propose a novel possibility based on the G4 perspective, but this hypothesis will require further experimental verification.*" (line 174 to 178)

"*Future studies could explore whether this variant contributes to non-syndromic cleft lip with cleft palate, possibly through its effect on G4 structure formation.*

Overall, G4SNVHunter can quickly identify variants that disrupt the formation propensity of G4 structures, thereby offering a structural perspective interpretation of non-coding variants for downstream analysis." (line 184 to 189)

Reviewer #2:

General comments: In this manuscript, Zhang et al. developed an R/Bioconductor package called G4SNVHunter for evaluating the impact of SNVs on G4s formation. This package is mainly based on G4Hunter, a widely used algorithm for G4 propensity prediction. Although this work does not introduce significant algorithmic improvements or innovations, the developed tool provides researchers with a convenient way to assess the impact of SNVs on G4s formation. Additionally, the authors have provided detailed instructions on the installation and usage of the tool, which adds value to their work. Overall, the manuscript is clear and easy to understand.

Response: Thank you for your appreciation and positive feedback.

However, I would like the authors to provide a more detailed explanation of the design of their tool, particularly the "Workflow of the G4SNVHunter package" section. For instance, it would be helpful to elaborate on the workflow of the tool, especially clarifying the relationships among different modules such as G4HunterScore, G4HunterDetec, GRanges, DNAStringSet, and SNVImpactG4. A more explicit framework diagram illustrating these relationships would be highly beneficial.

Response: We agree with your valuable suggestion.

We added a new flowchart (Figure 1B) to the revised manuscript, which clearly illustrates the calling relationships between core functions (e.g., *G4HunterDetect*, *G4VarImpact*). This diagram helps users to understand the functional roles and coordination of different modules within the analysis workflow.

Figure 1A was retained as a schematic illustration showing the overall steps of the analysis process, helping readers grasp the tool's application scenarios from a global perspective.

To further improve the user experience and help users become more familiar with different functions, we have added a "Quick Start" section in vignette (https://rongxinzh.github.io/G4SNVHunter/G4SNVHunter.html), which allows users to quickly run a complete set of analysis process with a few lines of code. We believe these improvements will significantly enhance the tool's usability and clarity.
